# Polo Like Kinase 2 Tumour Suppressor and cancer biomarker: new perspectives on drug sensitivity/resistance in cancer

**DOI:** 10.18632/oncotarget.332

**Published:** 2012-01-28

**Authors:** Helen M. Coley, Eleftheria Hatzimichael, Sarah Blagden, Iain McNeish, Alastair Thompson, Tim Crook, Nelofer Syed

**Affiliations:** ^1^ Faculty of Health and Medical Sciences, University of Surrey, Guildford, Surrey GU2 7XH, UK; ^2^ Department of Hematology, University Hospital of Ioannina, Ioannina, Greece; ^3^ Department of Medical Oncology, Hammersmith Hospital, London, W12 0HU, UK; ^4^ Department of Medical Oncology, St Bartholomew's Hospital, London, EC1, UK; ^5^ Dundee Cancer Centre, University of Dundee, Ninewells Hospital, Dundee DD1 9SY, UK; ^6^ Neuro-Oncology Research Group, Charing Cross Hospital, Imperial College, London, W6 8RF, UK

**Keywords:** Polo Like Kinases, Chemotherapy resistance, collateral sensitivity

## Abstract

The polo-like kinase PLK2 has recently been identified as a potential theranostic marker in the management of chemotherapy sensitive cancers. The methylation status of the *PLK2* CpG island varies with sensitivity to paclitaxel and platinum in ovarian cancer cell lines. Importantly, extrapolation of these *in vitro* data to the clinical setting confirms that the methylation status of the *PLK2* CpG island predicts outcomes in patients treated with carboplatin & paclitaxel chemotherapy. A second cell cycle regulator, p57Kip2, is also subject to epigenetic silencing in carboplatin resistance *in vitro* and *in vivo*, emphasising that cell cycle regulators are important determinants of sensitivity to chemotherapeutic agents and providing insights into the phenomenon of collateral drug sensitivity in oncology. Understanding the mechanistic basis and identification of robust biomarkers to predict collateral sensitivity may inform optimal use of chemotherapy in patients receiving multiple lines of treatment.

## INTRODUCTION

The Polo Like Kinases (Plk) are a family of structurally similar but functionally (relatively) distinct mitotic kinases all of which share homology with the *Drosophila* Polo, mutants of which show defects in mitosis and meiosis. There are five human Plk which have critical roles in regulation of cell cycle and response to DNA damage. Whilst there is good experimental evidence that Plk1 may function as an oncogene and indeed is a leading target for developmental therapeutics in oncology, Plk2-Plk5 have properties more consistent with tumour suppressor genes [review 1]. Plk2 was initially identified as an early response gene in serum-starved cells re-challenged with serum, hence the original name Serum Inducible Kinase (Snk) [2]. Later it was shown that the gene is a transcriptional target of p53 and activates a G2-M checkpoint [3].

## PLK2 IS EPIGENETICALLY REGULATED IN HAEMATOLOGICAL NEOPLASIA

We used suppression: subtraction PCR to identify *PLK2* as a gene down-regulated in B lymphomas [4]. Remarkably, Plk2 was subject to methylation-dependent transcriptional silencing in 100% of Burkitt lymphoma (BL) cell lines and a similarly high proportion of primary BL. The CpG island was also methylated across the spectrum of B cell lymphomas and in a proportion of cases of B cell chronic lymphocytic leukemias (B-CLL), suggesting that the gene is frequently down-regulated in B cell dyscrasias. More recent studies from our groups have shown methylation in the *PLK2* CpG island in other haematological malignancies including multiple myeloma [5], acute myeloid leukemia (AML) and myelodysplastic syndromes (MDS) [6]. We found that the frequency of methylation was higher in MDS than in AML (88.4% vs 68.9%) and also was significantly higher in low and intermediate risk MDS compared to higher risk groups (p=0.04). Based on these findings we suggested that methylation of *PLK2* could be another mechanism that leads to increased apoptosis that characterizes early stage MDS [6]. Another finding in this study was a tendency for improved survival for both AML and MDS patients who displayed hypermethylation of the *PLK2* CpG island, suggesting that the gene may have a utility as a prognostic biomarker in myeloid malignancies. In multiple myeloma, the frequency of aberrant methylation was found to be 60%, and although no correlation was found between *PLK2* methylation and MTHFR genotypes, anemia, bone disease or advanced stage, a 48% lower risk of death was noted for patients with methylated *PLK2* CpG island [5].

## DYNAMIC EPIGENETIC CHANGES IN THE PLK2 CPG ISLAND METHYLATION WITH PACLITAXEL SENSITIVITY: A MODEL FOR DRUG RESISTANCE IN OVARIAN AND OTHER CANCERS?

The taxanes docetaxel and paclitaxel remain key chemotherapeutic agents in the treatment of many solid tumours, including breast cancer, epithelial ovarian cancer (EOC), non-small cell lung cancer and prostate cancer. Resistance to chemotherapy including to the taxanes remains, however, one of the most important factors limiting the ability of oncologists manage neoplastic disease long term. Whereas a subset of human tumours exhibit clinical hypersensitivity to chemotherapy [7], the majority typically respond transiently to cytotoxic drugs but ultimately acquire resistance leading to treatment failure and death. The time interval between initial response and clinical drug resistance varies widely. For example, small cell lung cancer demonstrates remarkable sensitivity to cisplatin and etoposide in the majority of patients but relapse is inevitable and frequently occurs within weeks of stopping chemotherapy. In EOC, the majority of cases are initially sensitive to first-line chemotherapy typically with carboplatin and paclitaxel, but in contrast to small cell lung cancer sensitivity may be retained for extended periods of time, with only gradual emergence of drug-resistance. It is standard clinical practice to re-challenge EOC patients with platinum-based chemotherapy if a disease-free interval of > 1 year has elapsed since previous exposure to platinum, but there are few other clinico-pathological parameters to guide the physician in the rational use of chemotherapy at first and subsequent relapses, underlining the importance of identifying and validating robust biomarkers of drug sensitivity / resistance.

An increasing consensus recognizes the importance of epigenetics (heritable non-structural changes in gene expression), as a major mechanism driving acquired resistance to chemotherapy [8]. A popular approach to identifying determinants of acquired resistance to anti-cancer agents is the use of cell lines made relatively resistant to cytotoxic drugs *in vitro* with subsequent analysis either via the testing of candidate genes or by expression profiling. We derived a series of novel EOC cell lines with acquired resistance to paclitaxel and carboplatin and showed that resistance to paclitaxel, often with cross-resistance to carboplatin, occurred with loss of the G2 checkpoint and apoptosis [9] and we asked whether this was associated with changes in expression of the Polo Like Kinases. Whereas expression of Plk1, Plk3 and Plk4 was unchanged, there was clear and reproducible down-regulation of Plk2 due to acquired methylation in the CpG-island at the 5' end of the gene. Increasing levels of resistance to paclitaxel correlated with incrementally decreased expression of Plk2 and increasing CpG island methylation. We propose that exposure to chemotherapeutic stress induces methylation in the CpG island of specific “resistance” genes and this seeding effect leads to increasing methylation as the level of resistance increases, a phenomenon previously described in cells exposed to 6-mercaptopurine [10]. A study by Matthew et al. [11] described an *in vivo* study that considered the influence of hypoxia on chemosensitivity in Plk2-deficient tumours. Complete resistance to camptothecin was shown which pointed to an interplay between Plk2 and tumour micro-environment, whereas in the same experiments normoxia was associated with increased drug sensitivity. In the same study, it was shown that Plk2 can inhibit mTOR signaling under the influence of wild-type p53 control.

**Figure 1:**
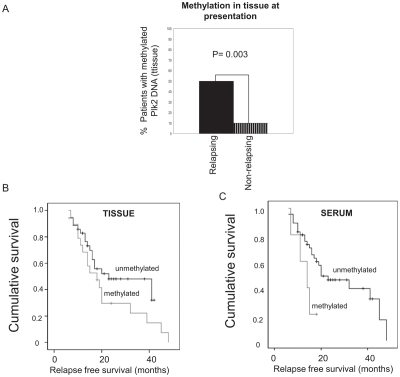
Methylation of Plk2 is associated with resistance to chemotherapy in EOC Genomic DNA was isolated from primary EOC tissue and matched serum. Methylation in the Plk2 CpG island was analysed by MSP as described in Methods. A: MSP analysis of Plk2 in tumour tissue from EOC cases at initial presentation; Solid black panel = patients with subsequent relapse; Shaded panel = patients who had not relapsed at the time of censor. p value given (Fisher's Exact test). B: Kaplan-Meier analysis of cases with and without Plk2 methylation in tissue at presentation and at relapse as indicated (p = 0.174, Log rank) and C: Kaplan-Meier analysis of cases with and without Plk2 methylation in serum at presentation and at relapse as indicated (p = 0.054, Log rank). Note follow-up period is censored if the patient is still alive at the time of the assessment. Censored data is indicated by (□).

In clinical cases of EOC, DNA methylation of the CpG island in the *PLK2* promoter in tumor tissue was associated with a higher risk of relapse in patients treated post-operatively with carboplatin and paclitaxel and this trend was also reflected in the analysis of matched serum samples in which detection of methylated *PLK2* genomic DNA was more frequent in relapsed cases [9], Figure 1A. Further, we were able to show in the same series that for patients for whom methylated PLK2 was detected in either or both tumour tissue and matched serum there was reduced survival, shown by Kaplan-Meier analysis (Figure 1B and 1C). Independent evidence of an important role for Plk2 as a determinant of chemotherapy sensitivity was afforded by a micro-array study which identified Plk2 as a down-regulated gene in chemotherapy-resistant EOC [12]. Plk2 was also independently identified as a critical determinant of clinical sensitivity of B CLL to chemotherapy, failure of Plk2 induction by fludarabine strongly predicting chemotherapy resistance [13]. Interestingly, when A2780 cells with high level resistance to paclitaxel were grown in the absence of maintenance exposure to the drug, a paclitaxel sensitive population re-emerged which re-expressed Plk2 due to loss of methylation in the *PLK2* CpG island. These proliferated at a higher basal rate than the paclitaxel-resistant cells from which they derived, an effect phenol-copied (at least in part) by ectopic expression of Plk2 [9] and unpublished observations). This was to the best of our knowledge the first demonstration that specific epigenetic changes are reversible when the selective pressure that initiated and then sustained evolution of a drug resistant cell population is removed. Our observations may partially answer the question of why the longer an individual cancer patient is not exposed to anti-cancer agents, the higher the probability that clinical response will be seen upon re-challenge with the original chemotherapy (a regular management strategy in platinum sensitive EOC and in other cancers treated with platinum such as non-small cell and small cell lung cancer and mesothelioma). In this model, methylation-dependent transcriptional silencing of key cell cycle regulators would increase with ongoing exposure to cytotoxic drugs and then diminish when chemotherapy is stopped. Re-expression of genes such as PLK2 and re-emergence of a more rapidly proliferating cell population would manifest as clinical relapse but with reacquired chemotherapy sensitivity.

## A WIDER ROLE FOR CELL CYCLE REGULATORS IN DRUG RESISTANCE IN OVARIAN CANCER

We have demonstrated a role for down-regulation of Plk2 as a pivotal factor underlying platinum / taxane resistance in EOC by a series of gene silencing experiments [9]. These findings prompt interesting questions concerning a possible more general role for cell cycle regulators as determinants of chemotherapy sensitivity/ resistance, consistent with work by other investigators [14]. Micro-array analysis of our own series of novel paclitaxel- and platinum-resistant EOC cell lines has revealed that several such genes are down-regulated in isogenic paclitaxel-resistant cell lines, including *Wee1* and *p57^Kip2^*. Down-regulation of p57^Kip2^ results in unopposed activity of the cyclin dependent kinases (CDK) and we hypothesized that this could confer collateral sensitivity to CDK inhibitors. Testing the CDK2 inhibitor seliciclib in EOC cell lines confirmed that this is indeed the case [15]. These results raise the intriguing possibility that a subset of EOC, in which acquired resistance to chemotherapy occurs via transcriptional silencing of cell cycle regulatory genes such as p57^*Kip2*^ are collaterally sensitized to pharmacological inhibition of the CDKs.

## COLLATERAL SENSITIVITY OF DRUG RESISTANT CELL LINES – ARE THERE OPPORTUNITIES FOR RESENSITISATION?

The term “collateral sensitivity” was first used in the field of microbiology to describe the “hypersensitivity” of otherwise antibiotic resistant E.coli (reviewed in Hall et al. 2009). The mechanistic basis underlying this phenomenon are varied and complex but nevertheless encompass a wide range of important chemotherapeutic drugs (reviewed in 16]. The concept that acquired resistance to one class of agent may confer “collateral sensitivity” to a second class of agents is established in the field of oncology and has been explored with a number of cytotoxic cancer chemotherapeutic agents [17], but has not been widely exploited in the clinical setting. Interestingly, it was shown that the collateral sensitivity of nitrogen mustard-resistant cells to chloroethyl nitrosoureas was attributable to the loss of guanine-O6-alkyl transferase activity present in the parent line, implying that changes in expression of specific genes confer both resistance and collateral sensitivity [18]. A study by Nakajima et al. [19] which examined two different time exposures of EOC lines to paclitaxel demonstrated a relationship between drug exposure time and extent of resistance induction, with collateral sensitivity to cisplatin. Another study by Xu et al. [20] analysed cisplatin-resistant gastric cancer lines generated by use of varying concentrations of inducing agent. Higher cisplatin-resistance was shown to correlate with collateral sensitivity to other platinum compounds, doxorubicin, mitomycin C and 5-FU. A variety of mechanisms associated with antioxidant and transporter genes was proposed by the authors [20]. There are also examples of platinum-resistance being associated with collateral sensitivity to anti-folate compounds [21].

Mechanistically, collateral sensitivity: resistance has been more rigourously tested with new targeted anti-cancer agents. Indeed, the literature cites a number of examples where new targeted therapy may be used advantageously in combination “cocktails”. Raf-1 transformed cells were relatively resistant to doxorubicin but collaterally sensitive to the Hsp90-targeting compound Geldanamycin [22]. The same study showed a combination of Geldanamycin and imatinib to be effective in BCR/Abl expressing cells, but only at unrealistic dose levels. An intriguing approach was to then consider the cell cycle inhibitor flavopiridol – which can act as a transcriptional inhibitor (as can the CDK inhibitor seliciclib) and could help to overcome the block in apoptosis seen in highly over-expressing BCR/Abl cells. Indeed, the triple combination of drugs enabled sensitization of the resistant cells to the effects of Geldanamycin [22]. Increased activation of AKT and /or a lack of PTEN is associated with tumours with a high proliferative response and chemotherapy resistance, but may confer sensitivity to mTOR inhibitors (reviewed in^23^]. This is further complicated by the fact that a feedback loop in the mTOR pathway leads to suppression of AKT and thus mTOR inhibition could actually lead to activation of AKT in some cells [24]. The *notch3, jagged* and *Wnt* genes have been shown to be expressed in serous ovarian cancers [25]. It is tempting to speculate that this tumour, which is notoriously chemorefractory, may be sensitive to specific targeted agents directed against such genotypes.

The EGFR directed tyrosine kinase inhibitor Gefitinib, whilst showing itself to be an effective therapy in the management of NSCLC with activating mutations in *EGFR*, is also associated with the development of resistance rendering it ineffective. NSCLC cell lines with acquired resistance to Gefitinib expressing reduced EGFR were shown to possess collateral sensitivity to TNF-alpha [26]. Even with reduced EGFR levels, Gefitinib resistant cells responded to TNF-alpha by autophosphorylation of EGFR with reduced AKT-phosphorylation.

Our observations, and those of other investigators, imply that a potential approach to therapy of solid tumours would envisage molecular profiling pre- and post-acquisition of clinical drug resistance. Transcriptional silencing of one or more key cell cycle regulators such as p57^Kip2^ would support the use of CDK inhibitors. Clearly, such an approach requires additional studies to determine the specific role of cell cycle regulators as determinants of resistance to individual chemotherapeutic agents and the effect of their loss, if any, in conferring collateral sensitivity to pharmacological inhibitors of the CDKs.

## CONCLUSIONS

Since the first description of anticancer drug resistance, investigators have identified a multiplicity of mechanisms which affect cellular sensitivity both to individual agents and more generally.

Alongside efforts to describe the mechanistic basis of reduced response to chemotherapy, there is a continuing requirement to identify robust biomarkers which predict patients at risk of developing chemotherapy resistant disease and suggest optimal alternative therapy for them. We have shown that the cell cycle regulators PLK2 and p57^Kip2^ are important determinants and candidate novel biomarkers of chemotherapy resistance in ovarian (and potentially other) cancers. Our results raise interesting considerations into collateral drug sensitivity in chemotherapy resistance both *in vitro* and in the oncology clinic.
